# Design and manufacturing of superabsorbent polymers (SAPs) for agriculture in arid areas

**DOI:** 10.1038/s41598-025-99666-6

**Published:** 2025-05-09

**Authors:** Maysa Muhammad, M. L. Tawfic, Mohamed Taha, Ahmed Elsabbagh

**Affiliations:** 1 Faculty of Engineering, Design and Production Engineering Department, Ain Shams University, Abbasiya, Cairo, 11517 Egypt; 2 Polymers Chemistry and Pigments Department, National Research Center (NRC), El Buhouth, Giza, 12211 Egypt

**Keywords:** Superabsorbent polymers (SAPs), Arid areas, Absorboost tile (AT), Hydrogel, Synthesis sodium Poly acrylate (NaPA), Synthesis Poly (sodium Acrylate-Co-Glycidyl Methacrylate) (AGMA), Rice straw, Sustainable agriculture, Environmental sciences, Engineering, Materials science

## Abstract

Innovative water-saving technologies aim to address the problem of water scarcity in agriculture, which faces significant challenges in solving the problem of agriculture in semi-arid and arid areas. This study specifically focuses on establishing a promising low-cost method to solve water scarcity and agriculture issues in semi-arid and arid areas. The objective of this study is to find an innovative engineering solution to design, manufacture, and produce an engineered product that has the potential to assist and change agriculture in semi-arid and arid areas. Design and manufacture of an innovative engineering product in the form of absorbent tiles (ATs), ATs can absorb and retain large volumes of water, helping to maintain moisture, and consistent moisture levels promote better root growth, and nutrient supply leading to healthier plants and increased crop yields, and reduce the need for frequent irrigation. The study objective was achieved by synthesis of two superabsorbent polymers (SAPs) materials, the synthesis of sodium polyacrylate (hydrogel#1) with five varying cross-linker content (wt.), synthesis of poly (sodium acrylate-co-glycidyl methacrylate) (hydrogel#2) with five varying cross-linker content (wt.), prepare the agriculture residual rice straw (RS) fiber length with three dimensions as a filler, prepare the natural adhesive (NA) solution as a biopolymer backbone, and design and manufacture hydraulic manual press ( test rig). These composites were mixed, compressed, and shaped to design and manufacture two tiles including AT1 (AT-NaPA) and AT2 (AT-AGMA) with different dimensions according to the effect root zone of the plant type to absorb and retain rainwater dew water and reduce irrigation frequency. Furthermore, ATs improve plant growth and nutrient supply under aridity condition and facilitate water conservation.

## Introduction

Agriculture is important for food production, economic development, sustainability, environment, and culture importance^1^. Water scarcity affects food security, health, and economic development. Addressing it requires integrated water resource management, sustainable practices, and technological innovations^2^. The problem statement of this study is divided into two categories, the first category is aridity in Egypt, and the second is water consumption management in agriculture. Egypt is mostly arid, covering about 96% of its area^3^. Egypt is divided into several arid areas such as the Nile Valley, Nile Delta, the Sinai Peninsula, and the western and eastern deserts^4^. The water consumption in Egypt in 2024 is 78 billion cubic meters. Agriculture is the largest consumer of water, accounting for about 78% of the total water use^5^. Water scarcity in plants seriously threatens agriculture in semi-arid and arid areas. Water shortages resulting from recurrent aridity can lead to reduced food production. These problems require solutions to manage the negative impact of aridity and water consumption in agriculture on crop production. The suggested approach is using the superabsorbent polymers (SAPs)^6,7^. Literature review for SAPs used for agriculture in powder morphology^8^for clay soil, SAPs are widely used in agriculture^6,7^to improve soil moisture retention and plant health. SAPs have higher water absorption, so these polymers are used to improve the soil water retention characteristics (WRC). Further, it is essential to determine the interactive effect of the water absorbency of water-absorbing polymers (WAP). The maximum WAP application concentration and plant available water content (PAWC) increase for coarse to fine soils ranged from 3.3- to 1.2-fold, respectively^9^. The development of fly ash-based water absorbing polymers (WAPs) for evaluating the growth performance of tomato varieties resulted in an increase in fruit number by 82%, total weight by 106%, and shoot biomass by 115%^10^. Here are some common types polyacrylamide-based SAPs are synthetic polymers that absorb and retain water efficiently, starch-grafted SAPs are made by chemically grafting synthetic polymers onto natural starch, offering a biodegradable alternative, cellulose-based SAPs are derived from plant cellulose, these polymers are environmentally friendly and biodegradable, polyvinyl Alcohol (PVA) SAPs are known for their water absorption capacity, these are used in agricultural soil amendments, chitosan-based SAPs are made from chitin (found in crustacean shells), these are organic and biodegradable, making them eco-friendly for sustainable farming, and natural rubber or protein-based SAPs are derived from natural materials and are gaining popularity for their biodegradability. The objective of this study is to design and manufacture absorboost tile (AT) to assists in the replacement of the sandy soil functions in semi-arid and arid areas, where the sandy soil has certain properties that make it less suitable for agriculture. The sandy soil features have large particles and wide spaces between them, which makes it unable to hold essential nutrients effectively, water drains quickly through sandy soil due to its coarse texture, leaving plants dry and prone to dehydration, sandy soil typically contains less organic material, which is essential for plant growth and overall soil fertility, and the loose texture of sandy soil can make it challenging for plants to establish strong roots, especially in high winds or heavy rains. The objective of this study is to solve these problems associated with the creation of an innovative engineering product (absorboost tile). Design, manufacture, and produce an engineering product in the form of an AT, as this AT assists in the replacement of the sandy soil functions in semi-arid and arid areas. For easy interchangeability, these AT dimensions are according to the effect root zone dimensions of the plant type. Another aim of ATs is durability, so they can be used for agriculture multiple times and have storage capability. The use of modern AT technologies for water conservation, improved soil health, improved crop yields, versatility, cost-effectiveness, eco-friendly, biodegradable, and environmental benefits. ATs have proven beneficial for growth and sandy soil health. NaPA (hydrogel#1)^12^and AGMA (hydrogel#2)^13^unique properties are associated with water conservation, improved soil health, improved crop yields, diversity, cost-effectiveness, environmental friendliness, biodegradability, and environmental conservation benefits. The advantage of AT is that it reduces water consumption by increasing infiltration and enhancing soil water holding capacity. ATs also protect the soil surface from temperature extremes and significantly reduce water evaporation, which is especially important in tropical and subtropical climates. ATs contribute to reducing soil degradation and water depletion in arid areas. In addition, ATs contribute to the impact of climate change by reducing the intensity of emissions and reducing losses from food and natural resources. The study objective was achieved by passing some steps. The first step is the synthesizing of two hydrogel materials used radical bulk polymerization process^11^, the synthesis of five samples of sodium polyacrylate (NaPA)^12^(hydrogel #1) with five varying cross-linker content (wt.), and the synthesis of five samples of poly (sodium acrylate-co-glycidyl methacrylate) (AGMA)^13^(hydrogel #2) with varying cross-linker content, (wt.). The second step is to prepare the ground rice straw (RS) specimens and prepare the natural adhesive solution. The third step is to design and manufacture a manual press (test rig) to produce AT. The fourth-step is the experimental analysis for ten samples synthesizing of two materials recipes (hydrogels) includes five different cross-linker content, (wt.) of sodium polyacrylate (NaPA), and poly (sodium acrylate-co-glycidyl methacrylate) (AGMA) according to American society for testing and materials (ASTM) standards. Evaluating their performance involves several analyses to assess their properties such as, gravimetric^14^include swelling capacity, absorption kinetics, gel fraction, water absorbency, protentional of hydrogen (pH)^15^, electrical conductivity (EC)^16^, and effect of temperature^17^. Structural characterization and morphological studies of SAP such as Fourier transform infrared (FTIR)^18^technique and scanning electron microscopy (SEM)^19^. The fifth-step is the technical processes for manufacturing AT-NaPA and AT-AGMA includes the NaPA or AGMA powder, RS preparation as a filler and choose the fiber length optimization, NA solution preparation as a biopolymer backbone, mixing, formulation, compacting and shaping. The sixth-step is the experimental analysis to evaluate the performance of AT according to ASTM standards and the food and agriculture organization (FAO). Evaluating their performance involves several analyses to assess their mechanical^20^, physical^21^, and chemical^22^properties such as, mechanical properties include compressive strength, gravimetric include absorption kinetic. Structural characterization and morphological studies of AT include Fourier transform infrared (FTIR) technique and scanning electron microscopy (SEM). Chemical properties include ions, organic matter, carbon-to nitrogen ratio, total nitrogen (N%), phosphorus (P%), and potassium (K%). Physical properties include bulk density, porosity, pH, and electrical conductivity, and finally pF curve^23^.

## Materials and methods

### Materials

Acrylic acid (AA purity > 98%), obtained from Marjan Chemical Factories, Abbasiya, Cairo, Egypt, was used as monomers. Sodium hydroxide, pellets (NaOH purity > 98%), obtained from Marjan Chemical Factories, Abbasiya, Cairo, Egypt, N, N’-methylene bis-acrylamide (MBAm or MBAA purity < 98%) is white crystalline powder,0.5% loss on drying, 0.02% AA, and 0.2% Al% aq 290 nm, obtained from Al-Gomhouria Pharmaceuticals Company, Amiri, Cairo, Egypt, was used as cross-linker. Potassium persulfate (K_2_S_2_O_8_purity > 98%) obtained from Marjan Chemical Factories, Abbasiya, Cairo, Egypt, was used as radical initiator, and glycidyl methacrylate (GMA purity > 99.8%) is Ether* 0.01%, appearance colorless and liquid, 1.073 density, and 1.450 refractive index at 20 °C, obtained from SIGMA-ALORICH, Spruce, Louis, USA, was used as monomers. Rice straw (RS) with an optimum moisture content^24^ of 8–12%, with the limitation of low lignin content, obtained from Mashtohor Toukh Village, Qalyubia, Egypt, was used as a filler. Distilled water was used in the preparation process.

### Synthesizing two materials recipes NaPA (hydrogel #1) and AGMA (hydrogel #2)

**Fig. 1:**
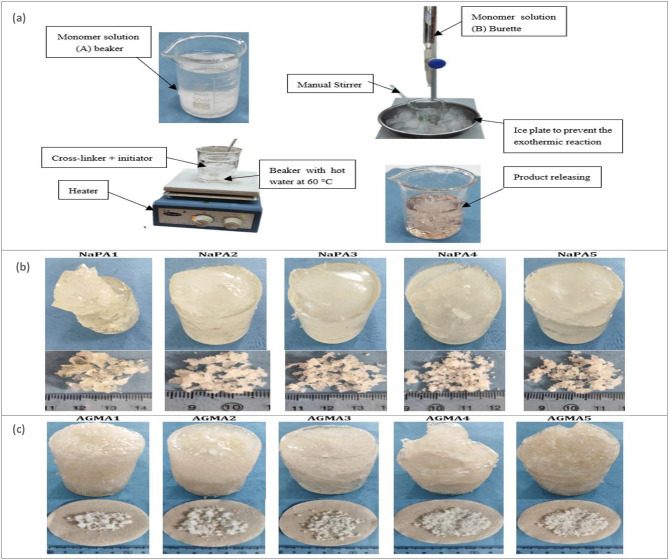
**a** Schematic representation illustrating the free radical bulk polymerization processes of NaPA (hydrogel #1) and AGMA (hydrogel #2). The steps of the process included a monomer solution beaker (**a**), a monomer solution burette (**b**), a hand stirrer, an ice plate, an ice plate to prevent the exothermic reaction, a cross-linker and initiator, water at 60 °C, a heater, the reaction temperature of 60 °C with a manual stirrer for 5:10 min. The product was placed in an oven at 60 °C for 24 h. **b** The pictures of the NaPA samples products released with five varying cross-linker content, (wt.), and NaPA samples with weight one gram after grinding. **c** The pictures of AGMA samples products released with five varying cross-linker content, (wt.), and AGMA samples with weight one gram after grinding.

Samples containing different amounts of AA (0.5 mol) as a monomer, MBA (1.3, 2.6, 3.9, 5.2, or 6.5 mmol) as cross-linker content for producing five samples to evaluate the effect of each sample on the best gravimetric analysis, NaOH (0.5 mol) solution as prepared using distilled water as the solvent, and KPS (3.7 mmol) as radical initiator. Figure 1a. shows the free radical bulk processes for prepare NaPA and AGMA. First, prepare NaOH solution by using distilled water (7–26 wt%) at 30 °C in a 100 mL beaker, determine the weight of (AA 0.5 mol) in the anther 150 ml beaker, and immerse it in an ice flask to prevent the exothermic reaction, that might lead to Michael addition reaction. Next, pour the NaOH solution into an (AA) beaker carefully adding dropwise with continuous stirring to ensure thorough mixing after finishing the NaOH solution and leaving the mixture of NaOH/AA for ten minutes. Then, place the mixture NaOH/AA in a hot bath at 60 °C while stirring. Finally, add wt. MBA, and wt. KSP to the NaOH/AA mixture with constant stirring (5–10 min) to maintain homogeneity until the hydrogel is transformed. The samples released were dried in an oven set at 60 °C, for 24 h, and put the samples in liquid nitrogen and grind for powders. The resulting products, a (hydrogel #1) referred to as NaPA with five different samples products released different cross-linker content, (wt.) was formed as shown in Fig. 1b. The resulting products, a (hydrogel #2) referred to the same synthesizing processes, but the different step added GMA to AA with ratio (1:1) as AGMA with five different samples products released samples were the pictures of the AGMA samples products released was formed as shown in Fig. 1c, and the NaPA or AGMA samples with weight (50–60) grams after grinding into powder.

## Design and manufacturing processes of AT production

**Fig. 2:**
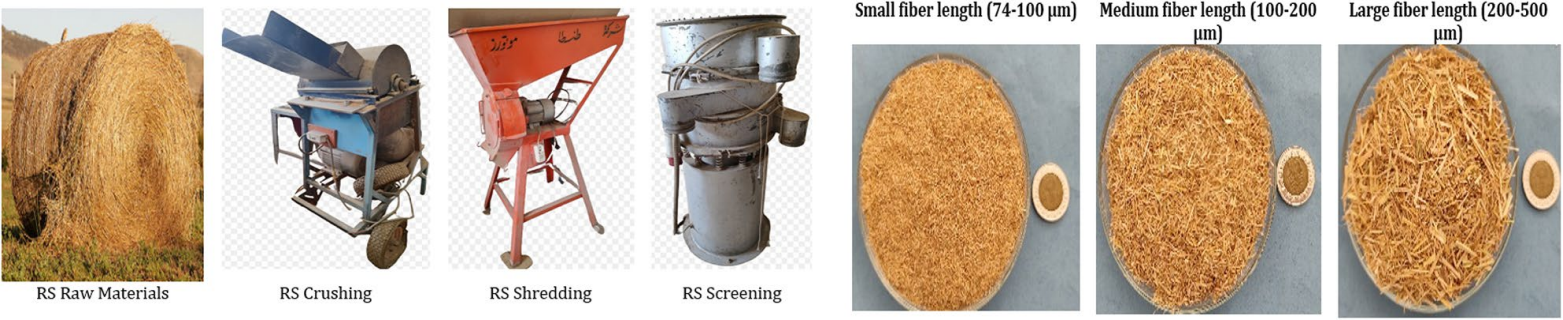
**a** Manufacturing processes of producing RS specimens, **b** The different fiber length dimensions of RS specimens.

**Fig. 3:**
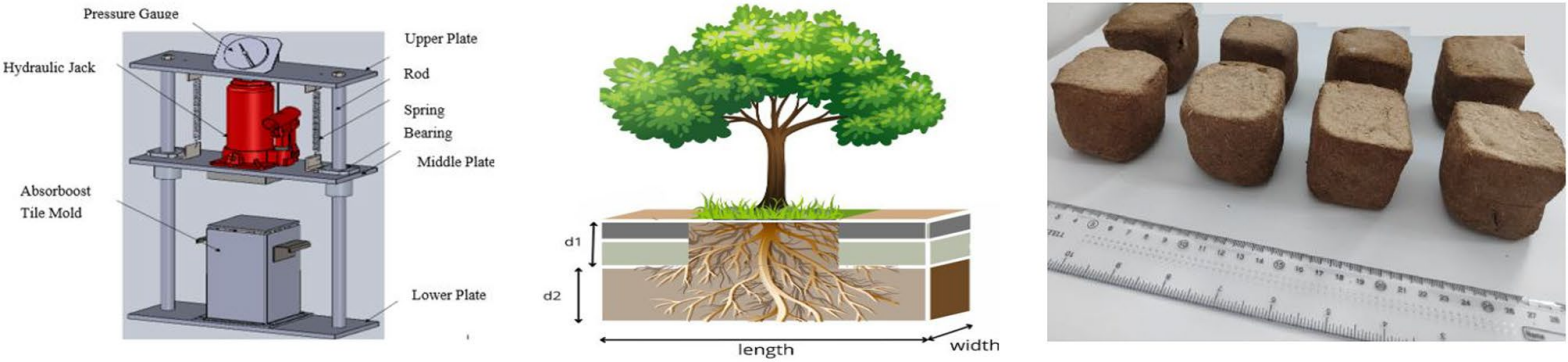
**a** A sketch of 3D drawing of the manual press used in the current work, **b** Effect root zone dimensions according to the plant type, and **c** AT dimensions, (5 cm* 5 cm* 5 cm) with four different weights of NaPA (hydrogel#1) and four different weights of AGMA (hydrogel#2).

The design and manufacturing processes of AT production were achieved by passing through some steps, the first step was synthesizing the five samples of NaPA (hydrogel #1) with different cross-linker content, (wt.) were used N, N’ methylene bis-acrylamide (MBA) with weights of (1.3, 2.6, 3.9, 5.2, or 6.5 mmol), and five samples of AGMA (hydrogel #2) with different cross-linker content, (wt.) were used N, N’ methylene bis-acrylamide (MBA) with weights of (1.3, 2.6, 3.9, 5.2, or 6.5 mmol), were synthesized based on a hygroscopic type (cross-linker) chemically prepared by free radical bulk polymerization. The second step is the preparation of RS samples, which are collected after milling the rice, dried to reduce moisture content, crushed on a crushing then shredded, and screened for different fiber lengths showed as Fig. 2a. The fiber length dimensions are classified as small rice straw particles (74–100 μm) and large rice straw particles (200–500 μm)^25^ showed as Fig. 2b. Small particles have a large surface area, and a high binder concentration strengthens the bonds between particles^26^. The third step is to design and manufacture a manual press (test rig) showed as Fig. 3a, where the dimensions of the mold depend on the dimensions of the effective root zone of the plant species showed as Fig. 3b. The fourth step is the optimum design of experiment (DOE) parameters of AT production using numerical methods and model techniques to analyze and optimize experimental parameters. DOE allows for the investigation of multiple factors simultaneously, reducing the number of experiments needed compared to one-factor-at-a-time approaches and saving time and resources. It helps identify which factors have the most significant impact on the response variable. In this study the response surface methodology (RSM) method, RSM is used to explore the relationships between several explanatory variables and one or more response variables. It helps optimize processes by fitting a polynomial model into the experimental data. The DOE parameters include RS fiber length (small 47–100 μm, medium 100–200 μm, or large 200–500 μm), natural adhesive (NA) (tapioca starch, gelatin, or risen), NA ratio (50%, 75%, or 100%), compaction pressure (low up to 5, intermediate 5–100, or high 100Mpa), the weight of NaPA or AGMA for concerning the weight of RS (2%, 3%, 4%, or 5%), cross-linker content, (1.3, 2.6, 3.9, 5.2, or 6.5 mmol) of NaPA or AGMA samples, porosity, and densification of RS (20%,35%, or 50%), temperature of NA (30 °C, or 50 °C). The fifth step is NA solution preparation and weight optimization. Finally, mixing, formulation, compacting and shaping of AT product dimension according to the effect root zone of the plant type. Figure 3c showed AT dimensions, (5 cm* 5 cm* 5 cm) with four different weights of NaPA and four different weights of AGMA. The weight of NaPA or AGMA samples is (2, 3, 4, or 5 g) for AT-NaPA1 or AT-AGMA1, AT-NaPA2 or AT-AGMA2, AT-NaPA3 or AT-AGMA3, and AT-NaPA4 or AT-AGMA4, respectively.

## Experimental

### Gravimetric of NaPA (hydrogel#1), AGMA (hydrogel#2) and AT samples

Gravimetric analysis was performed to optimize five NaPA (hydrogel #1), and five AGMA (hydrogel #2) powder samples, AT with four different weights of NaPA, and AT with four different weights of AGMA to describe the high-swelling after-powder synthesis and classify it into swelling capacity, absorption kinetics, gel fraction, and water absorbency.

Swelling capacity (%).

The swelling capacity of SAP materials refers to the amount of water or other liquid it can absorb. The experimental method according to ASTM C585-13. Calculate the percentage of the swelling capacity for NaPA and AGMA samples by the following equations:

$$\:\text{S}\text{C}\:\text{\%}=\frac{{\text{W}}_{2}-{\text{W}}_{1}}{{\text{W}}_{1}}$$

where:

W_1_: the weight of the SAP sample before swelling.

W_2_: the weight of the hydrogel sample after swelling.

Absorption kinetics (g/min) according to Fick’s First Law.

The absorption kinetics of SAPs describe the quickly of absorbed water or other liquid. Calculated the absorption kinetics for NaPA and AGMA samples by the following equation:

$$\:\text{A}\text{K}=\frac{{\text{W}}_{\text{g}}}{\text{T}}$$

where:

W_g_: the weight gain of SAP material samples every time interval.

T: time (minutes).

Gel fraction (g/g) and water absorbency (g/g).

The gel fraction of SAP refers to the properties of crosslinked formed within the hydrogel polymer network. Crosslinks enhance the mechanical strength and stability of hydrogel, and the gel fraction of hydrogel indicates a more stable and stronger hydrogel. The gel fraction hydrogel affects swelling capacity, resistance to dissolution, and mechanical strength. Calculate the gel fraction of SAP by the following equation:

$$\:\text{G}\text{F}=\frac{\text{W}\text{g}}{{\text{W}}_{\text{i}}}\:\text{x}\:100$$

where:

W_g_: The weight of hydrogel sample.

W_i_: The initial weight of the dried SAP sample.

The water absorbency of SAP defined the ability of absorb and retain of water or other liquid. Calculate the water absorption for NaPA and AGMA samples according to ASTM D570.

$$\:\text{W}\text{A}\:=\frac{{W}_{w}-\:{\text{W}}_{\text{d}}}{{\text{W}}_{\text{d}}}\:\text{x}\:100$$

where:

W_w_: The wet weight of hydrogel sample.

W_d_: The dry weight of SAP sample.

### Effect of potential of hydrogen (pH) and electrical conductivity (EC) of NaPA and AGMA samples

The pH test is a method used to determine natural, acidic, or basic (alkaline) a material and the pH ranges^27^ from 0 to 14. Agriculture applications testing soil pH to ensure optimal growing conditions for plants. The pH < 7 the material is acidic, the pH = 7 the material is neutral (like pure water), or the pH > 7 the material is basic (alkaline). The pH test measures the concentration of hydrogen ions (H^+^) in a solution, three methods for measurement effect of the pH such as litmus paper, pH meter, or indicator solutions. In this study used pH meter method. EC is used to evaluate the polymers interaction with water and its effectiveness in agriculture application, where maintaining optimal soil moisture and nutrient levels^28^.

### Effect of temperature (°C) of NaPA and AGMA samples

Temperature significantly affects the performance and properties of SAPs, such as swelling capacity, absorption rate, thermal stability, interaction with salts, and phase separation^29^.

### Structural characterization and morphological studies of NaPA, AGMA, and AT samples

Structural characterization and morphological studies of NaPA, AGMA, AT-NaPA, and AT-AGMA samples, such as Fourier transform infrared (FTIR) technique and scanning electron microscopy (SEM). The important of FTIR is identification of groups, structural analysis, quality control, monitoring chemical reactions, surface analysis, and degradation studies. SEM images illustrate the morphology of NaPA, AGMA samples before and after swelling, and AT with NaPA or AGMA samples are important for their performance. Measures the morphology according to ASTM D422. The shape of SAP fiber length and morphology of SAP and hydrogel are important for their performance.

### Mechanical, physical, and chemical properties of AT samples

Mechanical properties such as compressive strength analysis of AT with refers to the ability of a material or structure to withstand loads that tend to reduce its volume (compression), calculated according to ASTM D695. Measuring compressive strength involves determining the maximum load a material can withstand before failing. The physical properties of AT are divided into potential of hydrogen (pH), electrical conductivity (EC), bulk density, and porosity. pH stands for “potential of hydrogen” or “power of hydrogen” it is measure of the acidity or alkalinity of a solution. pH 7 is neutral (pure water), pH less than 7 indicates acidity, and pH greater than 7 indicates alkalinity (basicity). EC of AT is used to evaluate the polymers interaction with water and its effectiveness in agriculture application, where maintaining optimal soil moisture and nutrient levels. Bulk Density of AT is defined as the mass of dry soil per unit volume, including the air space, and it is indicator of soil health and compaction. Porosity of AT refers to the volume of pore spaces within the soil, and these pores are essential for the movement and storage of air, water, and nutrients, it is important for plant growth and soil health. Volume of pores indicates the proportion of the soil volume that is occupied by pores. High porosity means more space for air and water, promoting healthy root growth and microbial activity. The chemical properties of AT are divided into ions, and compound analysis, where compound analysis such as, organic matter content, carbon-to-nitrogen ratio (C: N), organic carbon, ash content, total nitrogen (N), potassium (K⁺) %, and phosphorus (P⁺) %. Ions includes the potassium (K⁺), sodium (Na⁺), calcium (Ca²⁺), magnesium (Mg²⁺), carbonate (CO₃²⁻), bicarbonate (HCO₃⁻), chloride (Cl⁻), and sulfate (SO₄²⁻).

### pF curve of AT samples

The pF curve also known as the soil-water retention curve describes the relationship between matric potential (a measure of soil moisture tension) and soil moisture content. The importance of pF curve measures of AT samples is water availability helps determine the amount of water available to plants at different soil moisture levels, which is crucial for understanding when and how much to irrigate, soil types different soils have different pF curves, which reflect their ability to retain and release water and knowing the pF curve for a specific soil type can guide agricultural practices, irrigation management comparing pF curves of different fields, farmers can decide which fields need irrigation first, and this helps in efficient water use and prevents waterlogging or drought stress, plant growth indicates the range between the wilting point (minimum moisture needed for plant survival) and field capacity (maximum moisture the soil can hold without waterlogging), and this helps in maintaining optimal soil moisture for plant growth.

## Results and discussions

### Gravimetric analysis

**Fig. 4:**
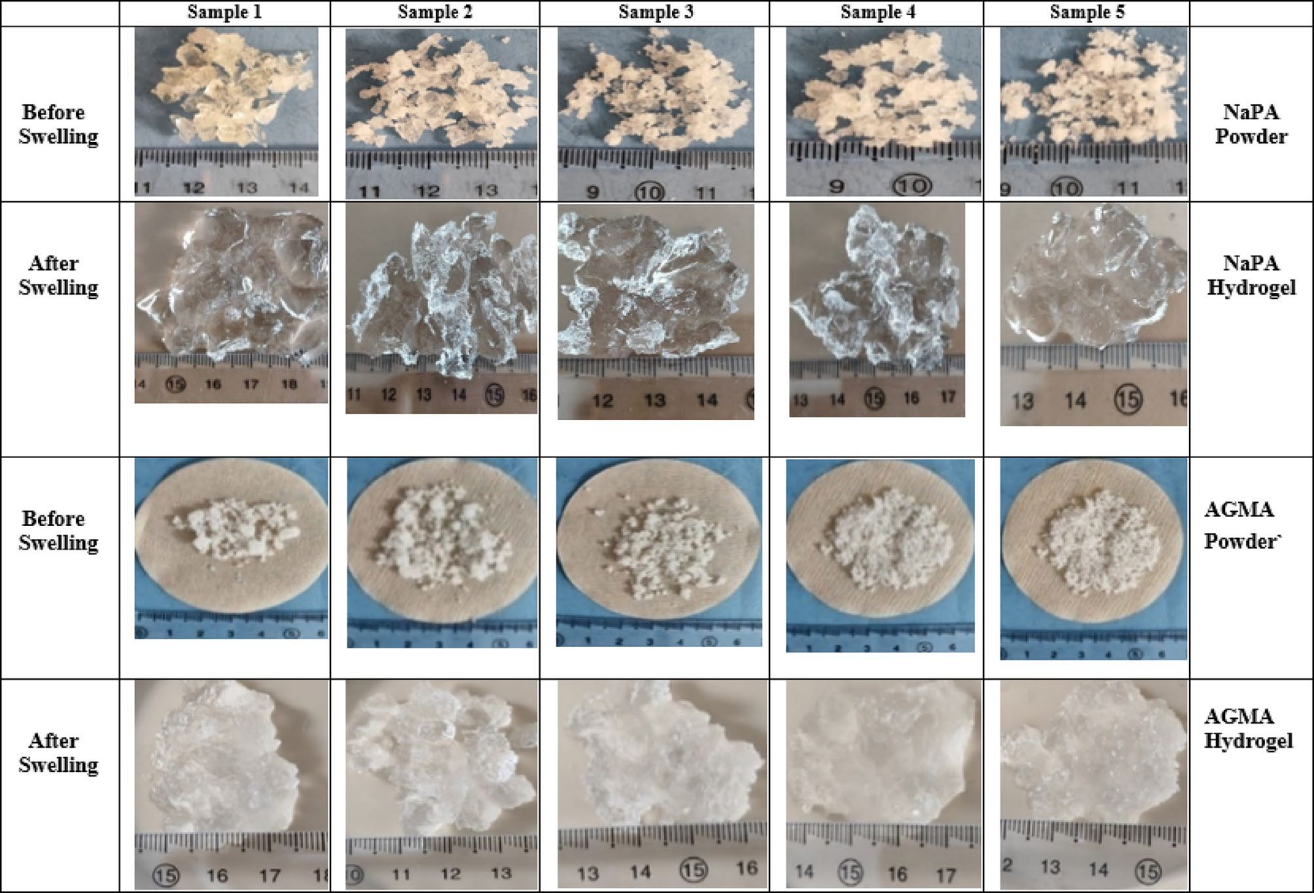
Schematic of the NaPA and AGMA hydrogel samples after swelling for hour in 100 ml distilled water with five varying cross-linker contents, (wt.). sample number one is the lower cross-linker content, (wt.) and gradually increasing the cross-linker by the following samples.

**Fig. 5:**
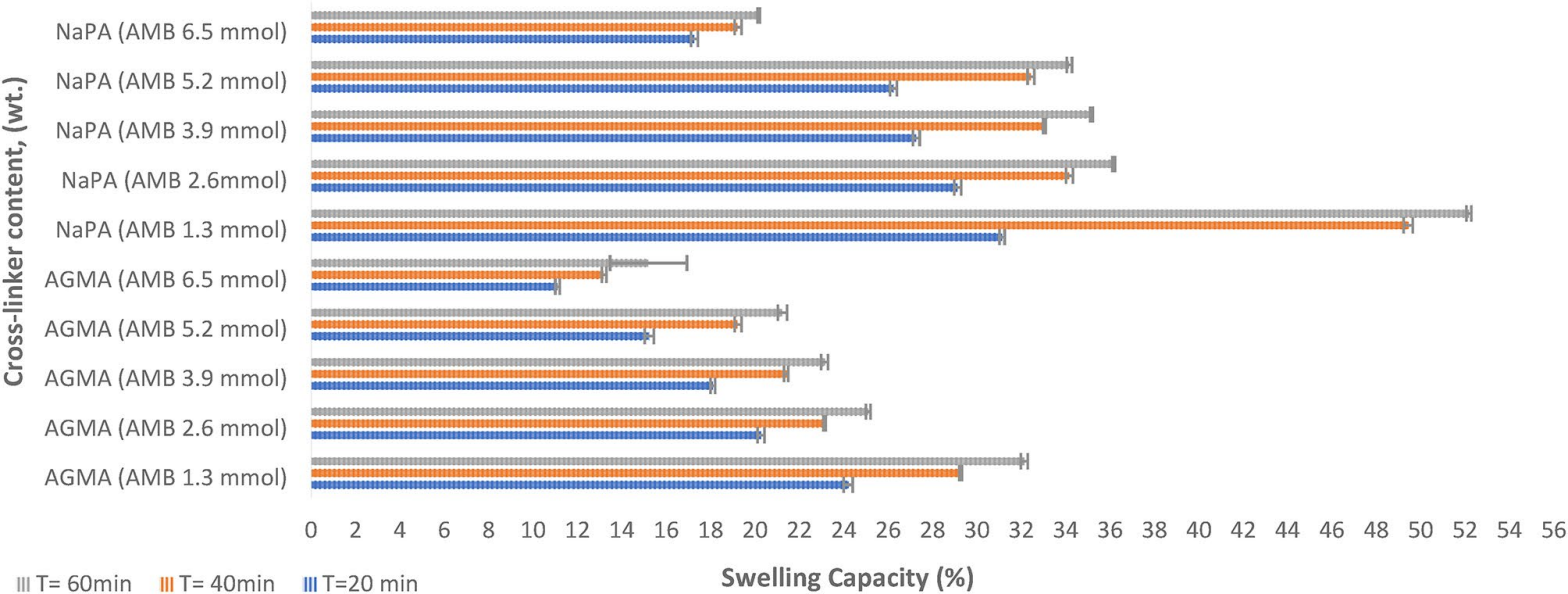
Swelling capacity (%) of NaPA (hydrogel#1) and AGMA (hydrogel#2) samples with five varying cross-linker content, (wt.) with time interval (min).

**Fig. 6:**
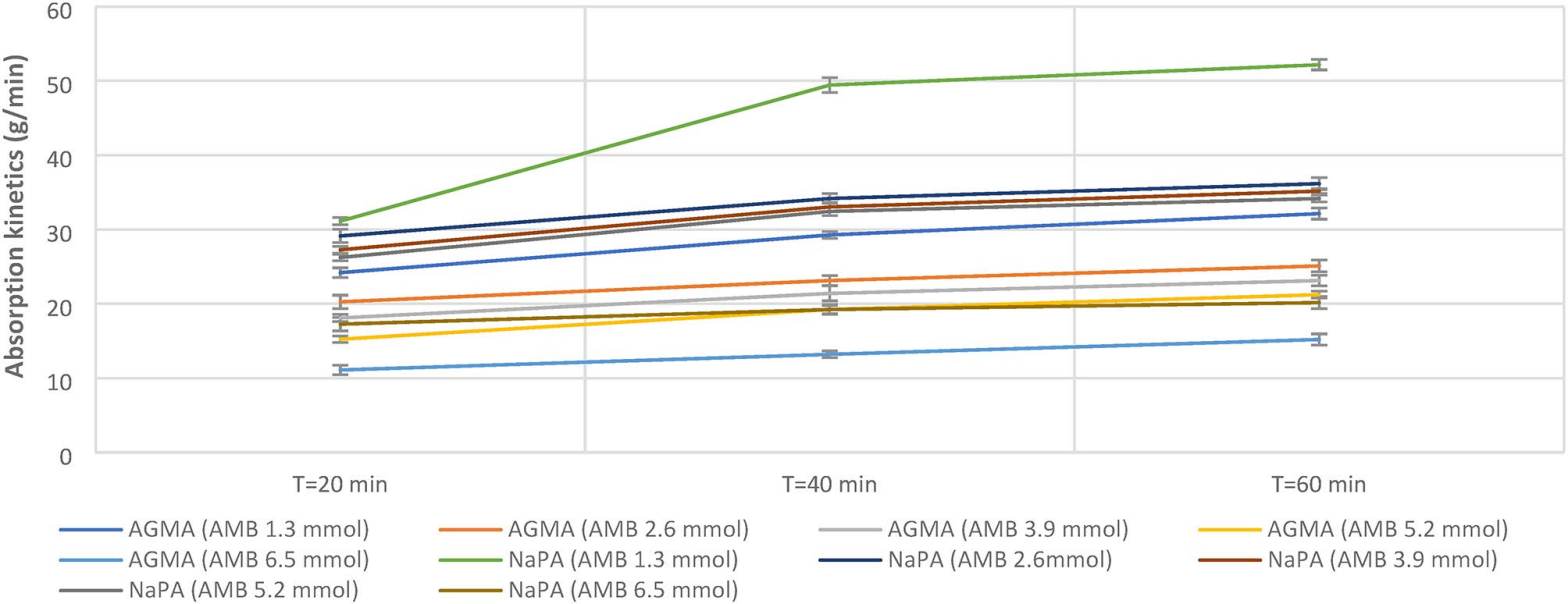
Absorption kinetics (g/min) of NaPA (hydrogel#1) and AGMA (hydrogel#2) with five varying cross-linker content, (wt.) with time interval (min).

**Fig. 7:**
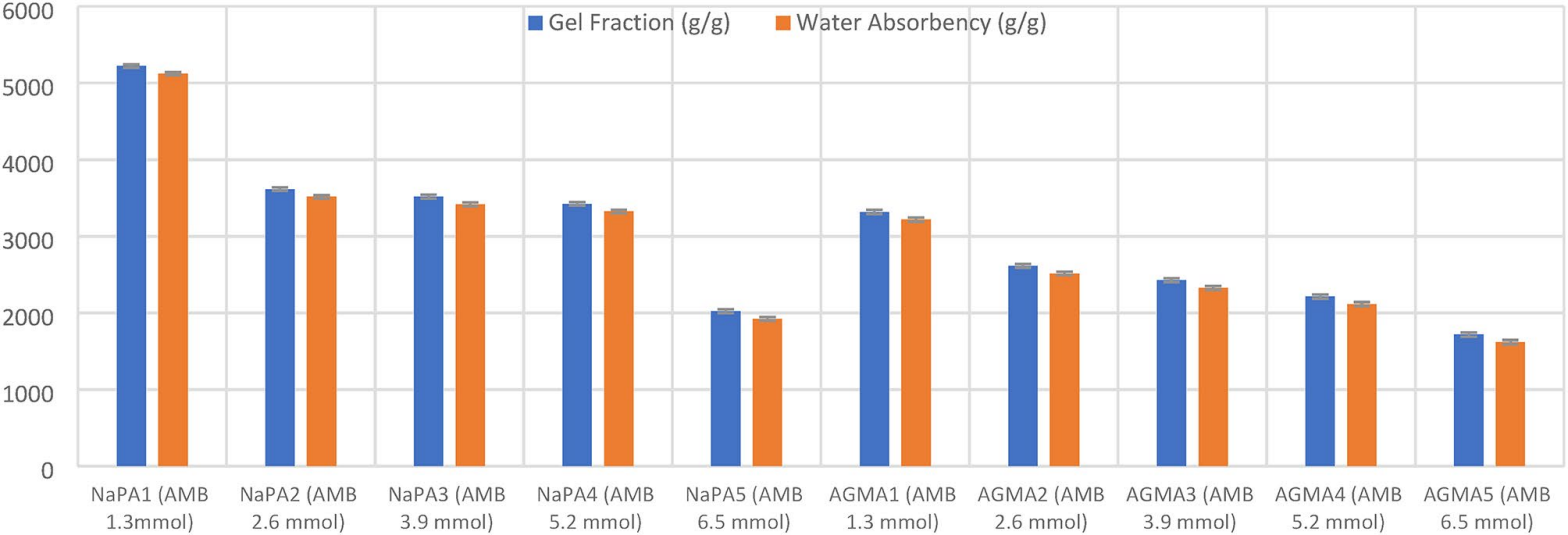
Gel fraction (g/g) and water absorbency (g/g) of five different cross-linker wt. of NaPA (hydrogel#1) and AGMA (hydrogel#2) samples with dry weight (100 ml).

**Fig. 8:**
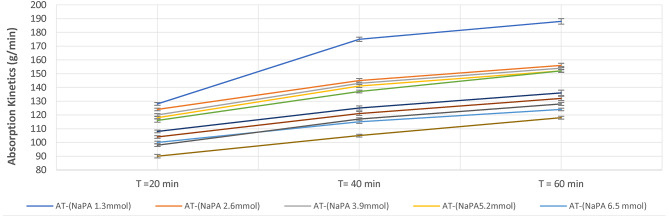
Absorption kinetics for AT-NaPA (AT1) and AT-AGMA (AT2) with five varying of cross-linker content, (wt.).

Figure 4 illustrates the pictures of ten samples for both NaPA (hydrogel#1) and AGMA (hydrogel#2) with initial weight one gram before and after swelling for hour in 100 ml distilled water with five varying cross-linker contents, (wt.). Sample number one is the lower cross-linker content, (wt.) (MBA 1.3 mmol) and increases the cross-linker by the following samples. Figure 5 illustrates the swelling capacity (%) of NaPA (hydrogel #1) and AGMA (hydrogel #2), two hydrogels synthesized with five different cross-linker contents, (wt.) with three-time intervals (min), and the experimental analysis of the swelling capacity is evaluated according to ASTM C 1585-13. By comparing the percentage of swelling capacity, the best results were obtained for five samples of NaPA hydrogel at a time of 60 min with low weight of MBA (1.3 mol) cross-linker content, (wt.). The percentage of the swelling capacity for (NaPA-MBA 1.3 mmol) is 51% at time 60 min, and the percentage of the swelling capacity for (AGMA-MBA 1.3 mmol) is 32% at time 60 min. Figure 6 illustrates the absorption kinetics (g/min) of NaPA (hydrogel#1) and AGMA (hydrogel#2) synthesized with five varying cross-linker contents, (wt.) with three-time intervals (min), and the experimental analysis of the absorption kinetics (g/min) is evaluated according to Fick’s first law. The graphical analysis between the wet weight of NaPA (hydrogel#1) or AGMA (hydrogel #2) five samples with varying cross-linker content gain up the y-axis against time and the slope of the curve illustrates the absorption kinetics. By comparing the values of NaPA or AGMA hydrogel five samples, the best result obtained for (NaPA1) (hydrogel #1) and (AGMA1) (hydrogel #2) with the low weight of cross-linker content (wt.), followed by increased in cross-linker content, (wt.). Figure 7 illustrates the gel fraction values (g/g) and water absorbency (g/g) of NaPA (hydrogel#1) and AGMA (hydrogel#2) with dry weight 100 ml and synthesized with five varying cross-linker contents, (wt.) and the experimental analysis of the gel fraction is evaluated according to ASTM D2765-16 and water absorbency (g/g) is evaluated according to ASTM D570-98. By comparing the percentage of gel fraction and water absorbency 5200 (g/g) and 5100 (g/g) are the best result obtained for sample (NaPA-MBA 1.3 mmol) hydrogel at low weight of cross-linker content (mmol). By comparing the percentage of gel fraction and water absorbency 3300 (g/g) and 3200 (g/g) are the best result obtained for sample (AGMA-MBA 1.3 mmol) hydrogel at low weight of cross-linker content (wt.). The absorption kinetics (g/min) of AT manufactured with five varying cross-linker contents, (wt.) with three-time intervals (min), and the experimental analysis of the absorption kinetics (g/min) is evaluated according to Fick’s first law. The graphical analysis between the wet weight of AT-NaPA and AT-AGMA five samples with varying cross-linker content, (wt.) gain up the y-axis against time and the slope of the curve illustrates the absorption kinetics. By comparing the values of AT- NaPA and AT- AGMA five samples, the best result obtained for AT1-(NaPA-MBA 1.3 mmol) and AT2-(AGMA-MBA 1.3 mmol) with the low weight of cross-linker content (wt.). Figure 8 illustrates the absorption capacity (g/min) for AT-NaPA and AT-AGMA with five varying of cross-linker content, (wt.). Finally, the optimal sample with the low cross-linker content, (wt.). sample1 and following the samples by gradually increasing cross-linker content, (wt.).

### Potential of hydrogen (pH) and electrical conductivity (EC) of NaPA (hydrogel #1) and AGMA (hydrogel #2)

**Fig. 9:**
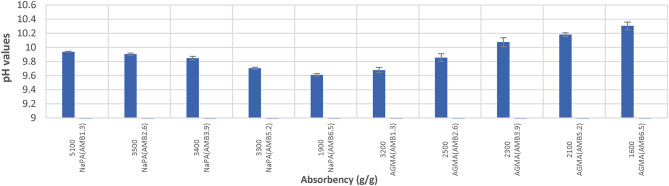
pH values for water absorbency (g/g) of NaPA (hydrogel#1) and AGMA (hydrogel#2) samples.

**Fig. 10:**
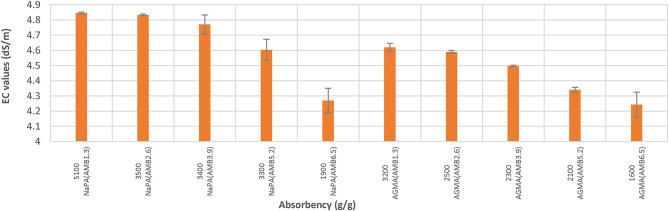
EC (dS/m) values for water absorbency of NaPA (hydrogel#1) and AGMA (hydrogel#2) samples.

The pH < 7 the material is acidic, pH = 7 the material is neutral, or the pH > 7 the material is basic. Figure 9 illustrates pH values for water absorbency of NaPA and AGMA hydrogels samples. Figure 10 illustrates EC (dS/m) for water absorbency of NaPA (hydrogel#1) and AGMA (hydrogel#2) samples. By comparing pH and EC, the optimal results from (9–10) for NaPA and from (9–11) for AGMA hydrogels samples after hour swelling in 100 ml distilled water obtained for five cross-linker content, (wt.). Electrical conductivity (EC) of hydrogels refers to the ability of the polymer to conduct electricity when in an aqueous state. The EC unit is measured in deciSiemens per meter (dS/m), EC is used to evaluate the polymers interaction with water and its effectiveness in agriculture application, where maintaining optimal soil moisture and nutrient levels. The EC (dS/m) of NaPA and AGMA hydrogel samples after hour swelling in 100 ml distilled water. The EC optimal results of NaPA and AGMA hydrogel samples (4–8) dS/m are moderately saline.

### Effect of temperature (°C) of NaPA (hydrogel#1) and AGMA (hydrogel#2)

**Fig. 11:**
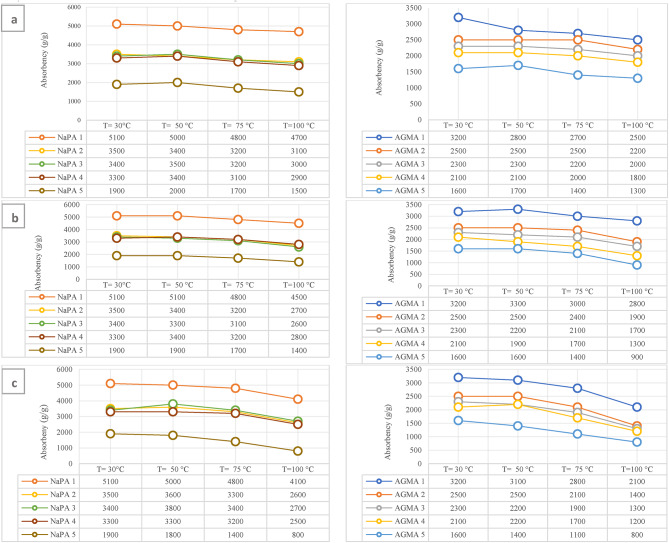
Absorbency of NaPA (hydrogel#1) and AGMA (hydrogel#2) samples under effect of temperature for different conditions (30, 50, 75, or 100 °C) after three experimental analyses at (a) 2 h., (b) 4 h. or (c) 6 h.

Temperature is an important factor affecting the absorbency capacity of hydrogels. Different swelling behaviors were observed of NaPA and AGMA hydrogel samples after an hour of swelling, the absorbency of NaPA and AGMA samples decreased with temperature influenced by temperature. Figures 11illustrates the change in absorbency under different conditions (30, 50, 75, and 100 °C) of the effect of temperature of NaPA (hydrogel#1) and AGMA (hydrogel#2) samples after three experimental analyses at 2, 4, or 6 h for three different zone of temperature (30, 50, 75, and 100 °C). The first condition at time 2 h., next condition at time 4 h., and the last condition at time 6 h. The start temperature with NaPA and AGMA hydrogel samples at 30 °C the polymer network formed hydrogen bonds with the water molecules, developing a hydration shell around the hydrophilic group which enhanced water uptake capacity to a maximum absorbency (5100 g/g) for (NaPA-MBA1.3 mmol) and maximum absorbency (3200) for (AGMA-MBA1.3 mmol). Therefore, with increasing temperature, the absorption capacity decreased steadily due to the elasticity of the cross-linked polymer network, the optimum temperature range of hydrogels^30^ was 30–50 °C. Absorbency of NaPA and AGMA hydrogel samples under the effect of temperature after 2 h. The effect of temperature on the absorption (g/g) NaPA samples is 8% degradation at 2 h., and the effect of temperature on the absorption (g/g) AGMA samples is 22% degradation. Absorbency of NaPA and AGMA hydrogels samples under effect of temperature after 4 h. The effect of temperature on the absorption (g/g) NaPA samples is 12% degradation at 4 h. The effect of temperature on the absorption (g/g) AGMA samples is 24% degradation at 4 h. Absorbency of NaPA and AGMA under effect of temperature after 6 h. The effect of temperature on the absorption (g/g) NaPA samples is 20% degradation at 6 h. The effect of temperature on the absorption (g/g) AGMA samples is 34% degradation at 6 h. The first sample has the lowest cross-linker content (by weight) (MBA 1.3 mmol) and the following samples increase in cross-linker content weight.

### Fourier transform infrared (FTIR) technique of NaPA, AGMA, and AT samples

**Fig. 12:**
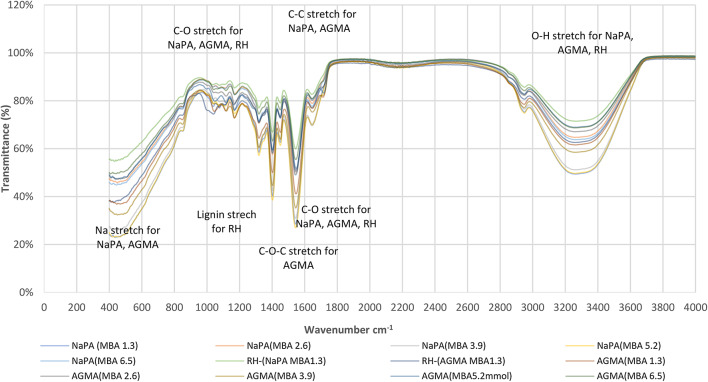
FTIR spectra for NaPA (hydrogel#1), AGMA (hydrogel#2) with five different cross-linker (wt.) and AT with NaPA (MBA 1.3 mmol) sample and AT with AGMA (MBA 1.3 mmol) samples.

FTIR transmission spectra illustrate the frequencies^31^ at which a sample absorbs infrared light. Figure 12 illustrates FTIR spectra of NaPA (hydrogel#1), AGMA (hydrogel#2) samples with five varying cross-linker content, (wt.) AT-1 (NaPA-MBA1.3 mmol), and AT-2 (AGMA-MBA 1.3 mmol) samples. The specific peaks and their intensities can vary depending on the sample composition and its molecular environment.

The FTIR transmission peaks of NaPA, and AGMA samples: (O-H stretch: water): The presence of oxygen and hydrogen ions inferred from absorption bands around 3200–3600 cm⁻¹. (C = C stretch double bond): The characteristic absorption of the C = C double bond in the acrylate monomer appears around 1600–1680 cm⁻¹. (C = O stretch the carboxylate group (COO⁻)): NaPA contains carboxylate groups, which illustrates a strong absorption band around 1400–1600 cm⁻¹. (C-O-C stretch: Epoxide group) Glycidyl methacrylate contains an epoxide group, which illustrates characteristic absorption around 1250–1000 cm⁻¹. (C-O stretch: carboxylic acids): NaPA contains carboxylic acids, which illustrates a strong absorption band around 1000–1100 cm⁻¹. (Sodium Ions (Na⁺) stretch): The presence of sodium ions can be inferred from the absorption bands around 400–600 cm⁻¹. The FTIR transmission peaks for RS samples: (O-H Stretch: Hydroxyl Groups): The presence of hydroxyl groups is indicated by absorption bands around 3422 cm⁻¹. (C = O Stretch: Carbonyl Groups): Absorption carbonyl groups, typically from hemicellulose and cellulose bands around 1740 cm⁻¹. (C-O Stretch: cellulose and hemicellulose): The presence of cellulose and hemicellulose bands around 1056 cm⁻¹. (Lignin): Lignin illustrates characteristic absorption bands around 1600–1500 cm⁻¹ and 1460 cm⁻¹.

### Scanning electron microscopy (SEM) of SAPs and AT

**Fig. 13:**
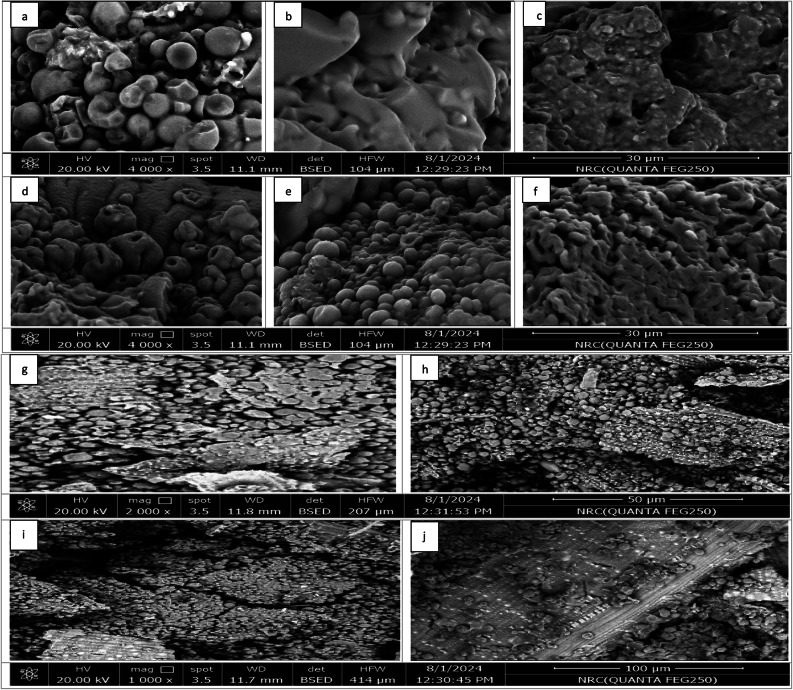
SEM images of NaPA and AGMA powders samples with three varying cross-linker content, (wt.) (**a**) (NaPA-MBA 1.3 mmol), (**b**) (NaPA-MBA 3.9 mmol), (**c**) (NaPA-MBA 6.5 mmol), (**d**) (AGMA-MBA 1.3 mmol), (**e**) (AGMA-MBA 3.9 mmol), (**f**) (AGMA-MBA 6.5 mmol), (**g**, **i**) AT1 (NaPA-MBA1.3 mmol), and (**h**, **j**) AT-2 (AGMA-MBA1.3 mmol) samples.

SEM images using thermal fisher scientific device to illustrate the morphology of NaPA (hydrogel#1), AGMA (hydrogel#2) with three different cross-linker content, (wt.) MBA (1.3, 3.9, and 6.5 mmol) and AT two sample includes, AT1-(NaPA-MBA1.3 mmol) and AT2-(AGMA-MBA 1.3 mmol). The method for describing these properties is morphology assessment. are important for evaluating their performance. The morphology measurement can be evaluated according to ASTM D422. Using image analysis, these techniques visually illustrate the round shape, porous, rough texture, and homogenous porosity. SEM images of swelling behavior illustrate by comparing SEM images before swelling, and can observe changes in the structure and understand how NaPA absorbs and retains water. Figure 13 illustrates SEM images for NaPA and AGMA powder samples with three varying cross-linker content, (wt.), the (NaPA-MBA 1.3 mmol) and (AGMA-MBA 1.3 mmol) samples have the lowest cross-linker content, the (NaPA-MBA 3.9 mmol) and (AGMA-MBA 3.9 mmol) samples have the medium content of cross-linker content, (wt.), and the (NaPA-MBA 6.5 mmol) and (AGMA-MBA 6.5 mmol) samples have the high cross-linker content, (wt.). By comparing the images for three categories of NaPA and AGMA samples illustrates the different shapes, homogenous porous, rough texture, different surface morphology, and illustrating these differential reflections on water absorption and retention. The results of the images are identical to the experimental analysis of NaPA (hydrogel #1) and AGMA (hydrogel #2) samples. SEM images of AT illustrate the morphology composites of RS as a filler, NA as a biopolymer backbone, and NaPA1 or AGMA1 samples are important for their matrix performance and the interaction. SEM images of two types of AT with (NaPA-MBA 1.3 mmol), and (AGMA-MBA 1.3 mmol) with the lowest and optimal cross-linker content. SEM images analysis can reveal the surface morphology and structure of RS, NA, and (NaPA-MBA 1.3 mmol) or (AGMA-MBA 1.3 mmol) composites, it illustrates the fibrous and porous structure of RS, rough surface, and the presence of silica and lignin, and SEM can reveal the granular structure of NA, with its characteristic crystalline and amorphous regions.

### Mechanical properties of AT

**Fig. 14:**
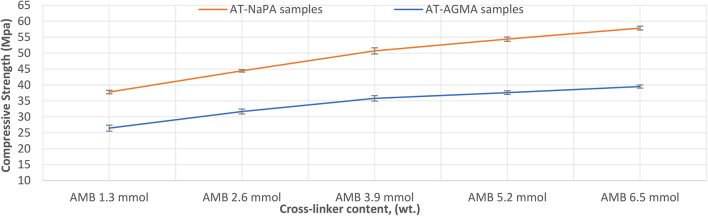
Compressive strength of AT-AGMA for five samples varying in cross-linker content, (wt.).

Compressive strength refers to the ability of a material or structure to withstand loads that tend to reduce its volume (compression), calculated as the compressive strength of AT according to ASTM D695. Figure 14 illustrates the compressive strength of AT with NaPA for five samples varying in cross-linker content, (wt.), the compressive strength results are 17.925 Mpa for (NaPA-MBA 6.5 mmol), 16.110 Mpa for (NaPA-MBA 5.2 mmol), 14.653 for (NaPA-MBA 3.9 mmol), 12.318 for (NaPA-MBA 2.6 mmol), and 11.967 for (NaPA-MBA 1.3 mmol). Figure 19 illustrates the compressive strength of AT with AGMA for five samples varying in cross-linker content, (wt.), the compressive strength results are 39.524 Mpa for (AGMA-MBA 6.5 mmol), 37.855 Mpa for (AGMA-MBA 5.2 mmol), 35.696 for (AGMA-MBA 3.9 mmol), 31.422 for (AGMA-MBA 2.6 mmol), and 26.527 for (AGMA-MBA 1.3 mmol). Finally, the maximum compressive strength for the higher cross-linker content, (wt.).

### Physical properties of AT

The physical properties of AT are divided into the potential of hydrogen (pH), electrical conductivity (EC), bulk density, and porosity. Table 1shows physical properties results and FAO^30^ standard values. AT samples with four different weights (2, 3, 4, or 5 g) of NaPA or AGMA with the optimal lower cross-linker content, (wt.) MBA (1.3 mmol). pH stands for “potential of hydrogen” or “power of hydrogen” which measures the acidity or alkalinity of a solution. pH 7 is neutral (pure water), less than 7 indicates acidity, and more than 7 indicates alkalinity (basicity). According to FAO, the suitable pH for soil is (6: 7.5), this range allows for the optimal availability of essential nutrients. pH values of AT for four different weights of NaPA or AGMA are alkaline soils (pH > 7.5). The EC results values of AT for four different weights of NaPA or AGMA are (4–8 dS/m). EC is a key indicator of soil salinity, according to FAO EC results of ATs are moderately saline. Porosity (%) is the volume of pores and indicates the proportion of the soil volume occupied by pores. High porosity means more space for air and water, promoting healthy root growth and microbial activity. According to FAO, porosity should be 50% of the soil volume, this space is necessary for storing air and water, these are essential for plant roots and soil organisms. The porosity results of ATs for four different weights of NaPA or AGMA are (70–80%). Bulk density is defined as the mass of dry soil per unit volume, including air space, and it is an indicator of soil health and compaction. According to FAO, low bulk density indicates good soil structure with ample pore space for air and water movement and is ideal for root growth and microbial activity. The bulk density values of AT for four varying weights of NaPA or AGMA are (0.23 g/cm^3^−0.32 g/cm^3^).

**Table 1:** Physical properties for Absorboost tile with four varying weights of NaPA and AGMA samples with the lower cross-linker content.

AT-sample	Bulk density (g/cm^3^)	pH	EC (dS/m)	Porosity (%)
FAO standard values	≺1	(6: 7.5)	(1–5 dS/m)	50
AT**-**1 = AT-NaPA (2 gram)	0.23	7.95	5.8	78
AT**-**2 = AT-NaPA (3 gram)	0.232	7.97	6.0	76
AT-3 = AT-NaPA (4 gram)	0.235	8.0	6.4	71
AT-4 = AT-NaPA (5 gram)	0.24	8.1	6.7	69
AT-5 = AT-AGMA (2 gram)	0.27	7.93	5.3	76
AT-6 = AT-AGMA (3 gram)	0.29	7.95	6.0	74.5
AT-7 = AT-AGMA (4 gram)	0.30	7.97	6.4	74
AT-8 = AT-AGMA (5 gram)	0.32	7.99	6.9	73

### Chemical properties of AT samples

Table 2Shows the food and agriculture organization (FAO)^32^ standard values (meq/L) of chemical ions of clay soil, with maximum and minimum suitable limits. The current study compares AT samples of four varying NaPA or AGMA to evaluate the quality of AT samples. The results of the experimental analysis are according to ASTM standards. The ion results of AT samples with NaPA and AGMA for eight samples of different weights with decreasing cross-linker content (by weight) are shown in Table 4. These results are significant for the limited quality of all AT samples. Ions are charged particles for soil chemistry and plant nutrition, are classified to cations and anions, where the soil cations. By comparison the potassium (K⁺), sodium (Na⁺), calcium (Ca²⁺), magnesium (Mg²⁺), carbonate (CO₃²⁻), bicarbonate (HCO₃⁻), chloride (Cl⁻), and sulfate (SO₄²⁻) experimental analysis results of AT with NaPA or AGMA samples concerning FAO lower and higher limits. AT samples are accepted to produce the interchangeability of soil functions in semi-arid and arid areas. The compound analysis of AT with NaPA (hydrogel #1) and AGMA (hydrogel #2) samples are important for plant health, fertility, and balancing these elements ensures robust plant growth and sustainable AT health. Table 5 lists FAO chemical standard^32^ for clay soil compound analysis. Table 5 shows compound analysis of AT samples with NaPA, or AGMA with four weights varying with lowest the cross-linker content, (wt.). Organic matter content (%) is enhances soil structure, water retention, and nutrient supply. Carbon-to-nitrogen ratio (%) indicates the balance between carbon and nitrogen in soil, impacting microbial activity and nutrient cycling. Organic carbon (%) essential for soil health, as it helps retain moisture, improves soil structure, and supports microbial life. Ash content (%) reflects the mineral content in soil after organic matter is burned off; helps in Understanding soil composition. Total nitrogen (%) crucial for plant growth, as nitrogen is a key component of amino acids, proteins, and chlorophyll. Potassium (K⁺ %) and phosphorus (P⁺ %) are vital nutrients for plant development. Potassium helps with water regulation and enzyme activation, while phosphorus is important for energy transfer and root development.

**Table 2:** FAO standard values for chemical ions for clay soil.

(FAO) Chemical standard for clay soil ions (meq/L)	Range	
	Max.	Min.
Potassium (K⁺)	2.0	0.1
Sodium (Na⁺)	85.0	9.0
Calcium (Ca²⁺)	10.0	1.0
Magnesium (Mg²⁺)	2.0	0.1
Carbonate (CO₃²⁻)	1.5	30.0
Bicarbonate (HCO₃⁻)	–	Less than 0.1
Chloride (Cl⁻)	13.0	3.0
Sulfate (SO₄²⁻)	6.0	1.0

**Table 3:** Ions values of ATs for NaPA and AGMA at four weights varying with lowest the cross-linker content, (wt.).

AT-sample	K^+^	Na^+^	Ca^+2^	Mg^+2^	HCO_3_^−^	CL^−^	SO_4_^−2^
AT**-**1 = AT-NaPA (2 gram)	1.81	33.5	6.2	4.51	0.3	40.7	5.0
AT**-**2 = AT-NaPA (3 gram)	1.95	39.6	6.5	4.35	0.34	45.3	5.09
AT-3 = AT-NaPA (4 gram)	1.21	43.6	6.9	4.30	0.42	49.7	5.29
AT-4 = AT-NaPA (5 gram)	2.5	52.0	7.3	4.20	0.5	59.1	5.4
AT-5 = AT-AGMA (2 gram)	2.0	35.6	8.1	4.30	0.3	39.1	5.75
AT-6 = AT-AGMA (3 gram)	2.07	46.1	7.9	4.47	0.32	51.2	6.31
AT-7 = AT-AGMA (4 gram)	2.19	52.3	7.3	4.71	0.37	59.9	6.91
AT-8 = AT-AGMA (5 gram)	2.3	61.3	7.4	5.00	0.4	68.2	7.4

**Table 4:** lists FAO chemical standard for clay soil compound analysis.

(FAO) Chemical standard of clay soil compound analysis	Range	
	Max.	Min.
Organic Matter Content %	5.0	0.5
Carbon-to-Nitrogen Ratio %	20:1	10:1
Organic Carbon %	3.0	1.0
Ash Content %	20.0	10.0
(Total) Nitrogen %	0.5	0.1
Potassium (K^+^ %)	2.0	0.1
Phosphorus (P^+^ %)	0.1	0.02

**Table 5:** Compound analysis for absorboost tiles for NaPA and AGMA with four weights varying with lowest the cross-linker content.

AT- sample	Organic Matter content %	Carbon-to- Nitrogen Ratio%	Organic Carbon %	Ash Content%	(Total) *N* %	K^+^ %	*P*^+^ %
AT-1	33.9	19.8	39.7	33.9	2.13	1.31	83
AT-2	40.1	19.6	39.1	34.01	2.19	1.35	84.8
AT-3	49.5	19.3	38.4	34.1	2.20	1.37	85.6
AT-4	55.8	19	38	34.2	2.23	1.4	87
AT-5	54.1	19.6	39.3	33.6	2.18	1.28	91
AT-6	54.7	19.67	39.33	33.9	2.21	1.31	87
AT-7	54.9	19.69	39.37	34	2.26	1.35	82
AT-8	55.3	19.7	39.4	34.1	2.29	1.37	79

### pF curve of AT samples vs. sandy soil

**Fig. 15:**
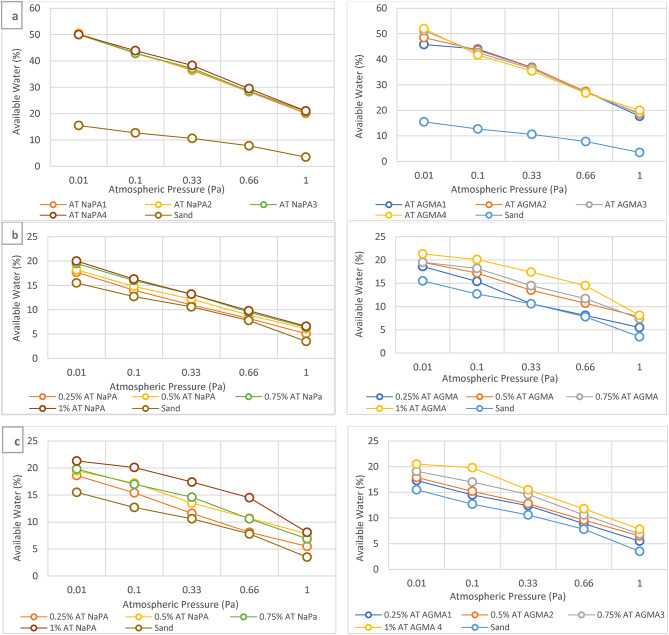
**a** pF curve of weight of AT-NaPA and AT-AGMA with four different weights Vs. sandy soil, **b** pF curve of weight of AT-NaPA and AT-AGMA with weight 2 gram Vs. sandy soil, and **c** pF curve of weight of AT-NaPA and AT-AGMA with weight 5 gram vs. sandy soil.

The pF curve, also known as a soil water retention curve, illustrates the relationship between the soil water content and the soil water potential expressed at available water capacity (AWC) at five values (0.01, 0.1, 0.33, 0.66, and 1) p.a. atmospheric pressure. Soil water content is the amount of water held in the soil at different potentials. Soil water potential (pF) is the energy of water in the soil, measured in centimeters of water column. The pF curve illustrates field capacity, permanent wilting point, and available water capacity (AWC). Field capacity is the amount of water remaining in the soil after excess water has drained away and the rate of downward movement has decreased, and this water is available for plant. Permanent wilting point is the point at plants can no longer extract water from the soil, leading to wilting. Available water capacity (AWC) is the range of water content between field capacity and the permanent wilting point, representing the water available for plant uptake. Figure 15a. pF curve of four different weight for AT-NaPA and AT-AGMA, where NaPA wt. or AGMA wt. (2, 3, 4, or 5 gram) with the optimal lowest cross-linker content, Vs. sandy soil at five values of atmospheric pressure (0.01, 0.1, 0.33, 0.66, and 1) p.a. Figure 15b. illustrates pF curve of four different weights of AT-NaPA or AGMA with 2 g from NaPA or AGMA, (0.25%, 0.5%, 0.75%, and 1%) with the optimal lowest cross-linker content addition to sandy soil Vs. sandy soil at five values (0.01, 0.1, 0.33, 0.66, and 1) p.a. atmospheric pressure. Figure 15c. pF curve of four different weights of AT-NaPA and AT-AGMA with 5 g from NaPA or AGMA, (0.25%, 0.5%, 0.75%, and 1%) with the optimal lowest cross-linker content addition to sandy soil Vs. Sandy soil at five values (0.01, 0.1, 0.33, 0.66, and 1) p.a. atmospheric pressure.

## Conclusions

The presented study successfully created an innovative engineering product that has the potential to assist and change the functions of sandy soil for agriculture and the limitation of the consumption of water for agriculture in semi-arid and arid areas. By synthesizing two biodegradable superabsorbent composites such as AT-NaPA and AT-AGMA were prepared NaPA and AGMA by free radical bulk polymerization processes of acrylic acid as monomers, potassium persulfate as initiator, N, N’ methylene bis-acrylamide as a crosslinker agent, natural adhesive as a biopolymer backbone and rice straw as filler. The effect of the first parameter of the concentration of the cross-linker content, (wt.), and the second parameter of the weight of NaPA (hydrogel #1) or AGMA (hydrogel #2) various components was investigated to individuate the optimal cross-linker content, (wt.) and optimal weight of NaPA or AGMA composition in terms of water absorption capacity, capable of controlled fertilizer release and enhanced water retention in soil. The optimized low cross-linker content (wt.) containing (1.3 mmol) of N, N’ methylene bis-acrylamide at swelling time 60 min showed a maximum water absorbency value in distilled water is (5100 g/g) and (3200 g/g) for (NaPA-MBA 1.3 mmol) and (AGMA-MBA 1.3 mmol), respectively. Furthermore, the RS and NA composites with (NaPA-MBA 1.3 mmol) or (AGMA-MBA 1.3 mmol) showed a maximum water absorbency value in distilled water at a swelling time of 60 min at (18,800 g/g) and (15,200 g/g), respectively. The swelling behavior of the prepared NaPA (hydrogel #1) and (hydrogel #2) was significantly affected by the nature of the external solution (pH and EC concentration). The pH results (9–11) are > 7 basic, and EC (4–8 dS/m) is moderately saline. The pH and EC results of AT1 (AT-NaPA) and AT2 (AT-AGMA) are obtained by adding RS and NA composites with NaPA or AGMA, the pH results decrease and the EC results increase. Temperature is an important factor affecting the absorbency capacity of NaPA and AGMA samples, swelling behaviors were observed NaPA and AGMA samples after an hour of swelling, the absorbency of NaPA and AGMA samples decreased influenced by temperature. The first condition at time 2 h. and at 30 °C the polymer network formed hydrogen bonds with the water molecules, developing a hydration shell around the hydrophilic group which enhanced water uptake capacity to a maximum absorbency (5100 g/g) for (NaPA-MBA 1.3 mmol) and (3200) for (AGMA-MBA 1.3 mmol). Therefore, with increasing temperature, the absorption capacity decreased steadily due to the elasticity of the cross-linked polymer network and the optimum temperature range for hydrogels in this study was 30–50 °C. The composites composed of hydrogels, RS, and NA exhibited exceptional water retention properties, and swelling capabilities, enhanced and improved soil structure, and nutrient supply, indicated the balance between carbon and nitrogen in the soil, supported microbial life, reflected the mineral content in the soil, crucial for plant growth, nutrients for plant development, and important for energy transfer and root development attributed to their chemical properties such compound analysis includes organic matter content, carbon-to-nitrogen ratio (C: N), organic carbon, ash content, total nitrogen (N), potassium (K⁺) %, and phosphorus (P⁺) %. The application of AT-NaPA or AT-AGMA effectively controlled release profiles showed an initial release followed by a continuous and gradual release of the base fertilizers (phosphorus (P⁺) %, potassium (K⁺), sodium (Na⁺), calcium (Ca²⁺), magnesium (Mg²⁺), carbonate (CO₃²⁻), bicarbonate (HCO₃⁻), chloride (Cl⁻), and sulfate (SO₄²⁻) over an extended period, allowing for prolonged delivery of nutrients to plants. Together, these features represent a significant advance over current water saving technologies in agriculture in terms of performance, reliability and practicality in sustainable agriculture.

## Data Availability

“Sequence data that support the findings of this study have been deposited in the Egyptian Intellectual Property (EGIPA) accession No. (EG/P/2024/001359), and Patent Cooperation Treaty (PCT)- World Intellectual Property Organization (WIPO) accession No. (PCT/EG2025/050012)”. (Data available with the paper or supplementary information: The authors declare that the data supporting the findings of this study are available within the paper and its Supplementary Information files. Should raw data files be needed in another format, they are available from the corresponding author upon reasonable request). All data generated or analyzed during this study are included in this published article or the Supplementary Information File.
